# Microarray and Functional Pathway Analyses Revealed Significantly Elevated Gene Expressions Associated with Metabolic Resistance to Oxamyl (Vydate) in *Lygus lineolaris*

**DOI:** 10.3390/toxics12030188

**Published:** 2024-02-28

**Authors:** Yu-Cheng Zhu, Yuzhe Du, Xiaofen Liu, Maribel Portilla, Jian Chen, Yanhua Wang

**Affiliations:** 1 United States Department of Agriculture, Agricultural Research Service, Jamie Whitten Delta States Research Center, Stoneville, MS 38776, USA; yuzhe.du@usda.gov (Y.D.); fanny.liu@usda.gov (X.L.); maribel.portilla@usda.gov (M.P.); jian.chen@usda.gov (J.C.); 2 Zhejiang Academy of Agricultural Sciences, Hangzhou 310021, China; wangyanh79@hotmail.com

**Keywords:** microarray, pathway, esterase, P450, oxidase, oxamyl, resistance, tarnished plant bug (TPB), *Lygus lineolaris*

## Abstract

The tarnished plant bug (TPB, *Lygus lineolaris*) remains a major pest for a variety of crops. Frequent sprays on row crops, especially cotton, prompted resistance development in field populations. To maintain chemical control as an effective tool against the pest, knowledge of global gene regulations is desirable for better understanding and managing the resistance. Novel microarray expressions of 6688 genes showed 685 significantly upregulated and 1382 significantly downregulated genes in oxamyl-selected TPBs (Vyd1515FF[R]) from a cotton field. Among the 685 upregulated genes (participated in 470 pathways), 176 genes code 30 different enzymes, and 7 of the 30 participate in 24 metabolic pathways. Six important detoxification pathways were controlled by 20 genes, coding 11 esterases, two P450s, two oxidases, and three pathway-associated enzymes (synthases, reductase, and dehydrogenase). Functional analyses showed substantially enhanced biological processes and molecular functions, with hydrolase activity as the most upregulated molecular function (controlled by 166 genes). Eleven esterases belong to the acting on ester bond subclass of the 166 hydrolases. Surprisingly, only one GST showed significant upregulation, but it was not involved in any detoxification pathway. Therefore, this research reports a set of 20 genes coding 6 enzyme classes to detoxify a carbamate insecticide oxamyl in Vyd1515FF. Together with three previous reports, we have obtained the best knowledge of resistance mechanisms to all four conventional insecticide classes in the economically important crop pest. This valuable finding will greatly facilitate the development of molecular tools to monitor and manage the resistance and to minimize risk to environment.

## 1. Introduction

The tarnished plant bug (TPB), *Lygus lineolaris*, feeds on a variety of crops and may cause serious economic loss. In cotton, both nymphs and adults pierce into plant tissues, secrete saliva (containing many digestive enzymes) for extra-oral digestion, and suck back plant juice. The feeding damages to cotton cause the dropping of squares and young balls, yield loss, and quality decrease [1]. Currently, management of TPB populations relies heavily on chemical insecticides. Thirteen insecticides have been recommended by Insect Control Guide Committee of Extension Service, Mississippi State University (2022, Publication 2471) for controlling TPBs on cotton, including pyrethroids, organophosphates, carbamates, neonicotinoids, and novel insect growth regulators.

Possessing systemic activity makes carbamate insecticides, such as Vydate (oxamyl), effective for suppression of TPBs with piercing/sucking mouthparts. Carbamates inhibit acetylcholinesterase (AChE) and poisoned bugs become hyper and eventually dead [2]. Average 4.75 sprays/year, plus additional 7–8 sprays for controlling non-sucking insects, are applied to control TPBs on cotton in the Delta area of Mississippi [3]. The long-term use of chemical insecticides for TPB control gradually decreased efficacy of insecticides and resistance to oxamyl has been found in many TPB populations in the mid-south [4,5]. Rosenheim [6] reported a case study and indicated that 27% of the chemical controls of closely related western tarnished plant bug (*L. hesperus*) using oxamyl may be categorized as “minor control failure” (population suppression rates were between 20% and <50%). “Major control failure” (suppression rate was <20%) happened when pyrethroid insecticide was applied [6].

Frequent insecticide exposures are the driving force for the resistance development in TPB populations. Small scale characterizations of a few individual detoxification genes and enzymes (P450s, esterases, and glutathione S-transferases (GST)) provided evidence for associating metabolic detoxifications closely with pyrethroid-, organophosphate-, and neonicotinoid-resistant TPB field populations [3,7,8,9,10,11,12,13,14]. Large-scale (global) analyses of 6688 transcriptional responses of a field TPB resistant population to the selections with representative insecticides of three classes of conventional insecticides (organophosphate, neonicotinoid, and pyrethroid) provided solid evidences of metabolic detoxification enzymes-mediated insecticide resistance in TPBs, by evolving a particular set of genes (esterases, oxidases, P450s, GSTs), together with a few dehydrogenase, synthase, reductase, and transferase genes in corresponding detoxification pathways [15,16,17]. 

A search of the literature indicated that the resistance mechanism to oxamyl in TPB has not been well studied. Because both organophosphates and carbamates have the same mode of action on acetylcholinesterase (AChE) [2], limited research has found that resistance development due to heavy use of organophosphates may prompt cross-resistance development to oxamyl in TPB populations in the mid-south area [18]. To understand resistance mechanisms to carbamate insecticide, great attention has been focused on mosquitoes because of their role in transmitting diseases to humans. Weill et al. [19] clearly showed that a single mutation (G119S of the ace-1 gene) explains this high resistance in *Culex pipiens* and in *Anopheles gambiae*. By investigating cross resistance to organophosphates and carbamates. Aïkpon et al. [20] suggested that the involvement of the ace-1 mutation could not entirely explain *An. gambiae* resistance to carbamate and organophosphate, because of the low rate of ace-1R allele frequency found in the resistant population. They provided biochemical evidence to suggest that resistance is mediated by metabolic resistance with elevated level of GST, MFO, and NSE, concluding multiple resistance mechanisms with both ace-1R target insensitive- and metabolic detoxification-involved resistance mechanisms, supported by Olé Sangba et al. [21]. Further studies revealed greater oxidase and esterase activities in resistant populations where ace-1 was absent [22] and the key role of esterases in biodegradation of organophosphate, carbamate, and pyrethroid pesticides was emphasized by Bhatt et al. [23]. With a finding of new mutation N485I of acetylcholinesterase-1, Ibrahim et al. [24] still revealed the importance of the P450 *CYP6Z1* in conferring carbamate/pyrethroid cross resistance in a major African malaria vector. Although mutations of the sodium channel and AChE have been known in other hemipteran insects for pyrethroid and pirimicarb resistance, no target-site resistance has been reported in TPB populations [3]. Due to the economic importance of TPBs and the potential risk of chemical control failure, it is urgent to know why and how TPB populations evolved resistance to carbamates. 

Understanding the resistance mechanisms may lead to developments of biochemical and molecular markers for monitoring resistance in field populations and tools to suppress resistance enzymes and genes. In this study, we collected a TPB population from a cotton field and treated the bugs with formulation Vydate^®^ (oxamyl). Novel microarray technology was used to examine thousands of gene expressions (at the same time) responding to the insecticide treatment. Functional and pathway analyses of significantly upregulated and downregulated genes separately identified specific genes that are responsible for detoxification and resistance development to oxamyl. In addition to major common detoxification genes, revealing a large number of other metabolic enzyme genes, showing significant transcriptional responses to oxamyl treatment, may expand our knowledge of carbamate resistance mechanisms in TPB.

## 2. Materials and Methods

### 2.1. Laboratory Colony and Field Population of the Tarnished Plant Bug (TPB, Lygus lineolaris)

A laboratory colony, established from a collection of tarnished plant bugs from the Mississippi Delta area, has been maintained since 2005 without exposure to insecticide. This colony was used as a standard susceptible strain (LLMCK) [17]. We started with an active search of more than 40 locations from the 100 × 161 km very flat Delta area of Mississippi and Arkansas to find a resistant population of TPB. A modified Porter spray tower was used to simulate common control practice (field spray treatment) to control most crop pests, LC_99_ values of Vydate (formulation of oxamyl) against susceptible LLMCK was 447.96 mg/L. A resistant population was collected from a cotton field (5 km north of Stoneville, MS, USA) and spray treated with 4× volume of Vydate solution at 1515 mg/L. Survivals (Vyd1515FF with ~13-fold resistance) from Vydate treatment were subjected to the analysis and comparison of gene expression levels between Vydate susceptible and resistant populations using microarray.

### 2.2. cDNA Library Sequencing, Microarray Processing, and Functional and Pathway Analyses

Details of materials and methods were described by Zhu and Luttrell [16]. In brief, approximately 30,000 cDNA library clones were obtained and sequenced with a M13 forward primer on an ABI 3730XL sequencer. Sequences were assembled into 7446 unique contigs and singletons. Sequences were subjected to a similarity search for putative identity against protein and nucleotide databases of the GenBank (http://blast.ncbi.nlm.nih.gov/Blast.cgi accessed on 31 January 2024). cDNA sequences were submitted to Roche NimbleGen (Roche NimbleGen, Inc., Madison, WI, USA) for production of 72 K gene expression chips in 4-plex format. A 60-bp specific oligonucleotide was designed and synthesized as a probe. Approximately 35,000 probes (average of 5 probes per cDNA) were synthesized and printed on each gene expression chip.

Microarray analysis was processed using standard NimbleGen array protocols. Total RNA was extracted from adults using TriZol reagent (Invitrogen, Waltham, MA, USA). Double-strand cDNAs were synthesized using the total RNA extracted from Vyd1515FF or LLMCK and the SuperScript Double-Stranded cDNA Synthesis Kit (Invitrogen). Double-strand cDNA samples were labeled using a One-color DNA Labeling Kit and hybridized to the microarray chips. Microarray data were acquired and normalized [25] according to NimbleScan v.25 User’s Guide through Florida State University Microarray processing facility. 

ArrayStar^®^ software Version 17 (DNAStar, Inc., Madison, WI, USA) was used to analyze and compare microarray data between LLMCK and Vyd1515FF. Upregulated and downregulated genes (cDNAs) were separately subjected to sequence annotation and functional pathway analyses using step-by-step processing of Blastx, mapping, annotation, enzyme code, KEGG/Nagegg, functional, combined functional, and pathway analyses using commercial Biobam OmicsBox bioinformatics software (https://www.biobam.com, accessed 31 January 2024). 

## 3. Results and Discussion

### 3.1. Scatter Plot Comparison of Gene Expression Levels between LLMCK(S) and Vyd1515FF(R)

**Figure 1:**
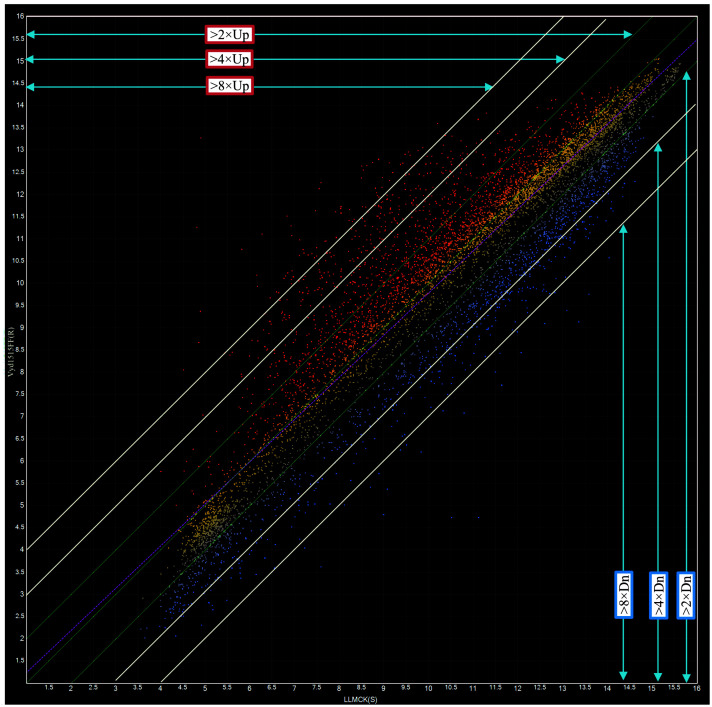
Microarray analysis using ArrayStar to reveal expression levels of 6688 genes between oxamyl (Vydate)-selected (Vyd1515FF(R)) and a susceptible (LLMCK(S)) tarnished plant bug colony. The scatter plot shows ratios of 6688 gene expressions of the resistant population against those of susceptible population. The mini squares in the upper left corner represented upregulated genes, and the squares in the lower right corner represented downregulated genes. Squares above line 2 × Up and below line 2 × Dn represent up and downregulated genes by 2-fold; Squares above line 4 × Up and below line 4 × Dn represent up and downregulated genes by 4-fold; Squares above line 8 × Up and below line 8 × Dn represent up and downregulated genes by 8-fold.

A total of 7446 unique contigs and singletons were obtained from cDNA library sequencing and 6688 genes had valid expression values from hybridization of labeled probs (double-stranded cDNAs of carbamate-resistant Vyd1515FF and susceptible LLMCK bug samples). Figure 1 was from scanned signals of hybridized gene chips of Vyd1515FF(R), that were log2 converted, normalized, and plotted against the corresponding gene signals of susceptible (LLMCK(S)) strain. The plot (Figure 1), generated by ArrayStar software, shows nearly normal distributions of 6688 gene expression levels. The location of each tiny square reflects a unique gene expression ratio between Vyd1515FF and LLMCK strain. A linear correlation (R^2^ = 0.878) exists between gene expression levels of the two strains. Statistics show the expressions levels of 2067 genes in oxamyl-selected (Vyd1515FF) bugs were up or downregulated by >2-fold (>log2× as significantly up or down), including 685 up and 1382 downregulated genes. Among the 2067 genes, 473 genes were up/downregulated by >4-fold and 96 genes were up/downregulated by >8-fold. Other 4621 (1662 up and 2959 down) genes were up/downregulated by less than 2-fold (Figure 1).

### 3.2. Identity, Abundance, and Potential Association of Upregulated Genes with Oxamyl Resistance

Among 685 upregulated (>2 fold) genes, 509 genes coded structure proteins, or their identities have not been identified. The rest of 176 genes code 30 different enzymes. Their sequence ID, sequence length, enzyme name, and the level (fold) of upregulation in oxamyl-selected TPB are listed in Table 1 and Supplementary Table S1. As many as 1382 genes were significantly downregulated in oxamyl-selected TPBs and 120 of those genes coded 29 different enzymes (Table 1 and Supplementary Table S2).

#### 3.2.1. Gene Expression Patterns of Major Detoxification Enzymes

P450 enzymes catalyze the oxidation of organic substances, degrade lipids, ecdysteroids and juvenile hormones, and metabolize xenobiotic substances of natural or synthetic origin [26]. In insects, P450 genes play a central role in the adaptation to plant chemicals and insecticide resistance development. It is well established that metabolic resistance to insecticides is associated with elevated levels of P450 gene expressions [24,27]. Six P450 genes (encoding cytochrome P450 monooxygenases, matching variable CYP families, Table 1) were significantly upregulated and no such a gene was downregulated in oxamyl-selected TPBs (Table 1 and Supplementary Table S1). An additional 10 P450 genes were all upregulated, although not significantly, in oxamyl-treated bugs. Our previous studies also found overexpression of P450 CYP6X1 in pyrethroid- and other P450s in organophosphate-resistant TPB populations [7,15], underscoring potential cross/multiple resistances existed in TPB, because P450s is a primary detoxification enzyme for many insecticides [28].

Eleven esterase genes were significantly upregulated and one (not involved in the detoxification pathway) was significantly downregulated in oxamyl-treated bugs. Additional five esterase genes did not show significant change in gene expression levels between susceptible and resistant strains. Esterases (or carboxylesterases) are frequently implicated in the resistance of insects to organophosphates, carbamates, and pyrethroids through gene amplification, upregulation, coding sequence mutations, or a combination of these mechanisms [8,29,30]. Esterases are also primary detoxification enzymes that cut ester bond-containing organophophate, pyrethroid, and carbamate insecticides into two parts via a hydrolyzing ester bond [31]. 

Only one GST (glutathione S-transferase) was significantly upregulated and another nine GSTs were not significantly upregulated in oxamyl-resistant TPB. The upregulated GST is not involved in detoxification pathway, although GSTs were associated with an organophosphate insecticide resistance in the TPB [9,32]. It is possible that most pathways were established from high animals. Further studies are necessay to validate its function in TPBs.

Most of the cytochrome oxidase genes (79%) were insignificantly downregulated. Only two and ten cytochrome c oxidase genes were significantly up and downregulated, respectively, in oxamyl-selected TPBs. Oxidase is a large transmembrane protein complex found in the mitochondrion. It is an oxidizing enzyme, catalyzing a redox reaction using dioxygen, as the terminal enzyme of the respiratory electron transport chain. Oxidase plays a vital role in ATP generation by oxidative phosphorylation. In a permethrin-resistant TPB population (the same population of Vyd1515FF was developed in this study), oxidase-involved oxidative phosphorylations were the major detoxification pathways [17]. Sixty-four oxidases (distributed in Subunits 1–3 and 6–10) and seven other oxidases (subunits 4–5 and 15) were significantly up and downregulated, respectively, in permethrin-resistant TPBs [17]. Cytochrome c oxidase Subunit 3 is the most abundant oxidase in permethrin-treated TPB followed by Subunits 7 and 2. Over expression of oxidase Subunits 3 and 1 was reportedly responsible for resistance development in many insects [33,34,35]. The two oxidase genes, significantly upregulated in Vyd1515FF and associated with detoxification pathways, belong to cytochrome c oxidase Subunits 1 and 15. None of these two oxidase genes were significantly upregulated in the permethrin-resistant TPBs [17], indicating they are specialized for detoxifying oxamyl, a carbamate insecticide. None of the 10 other significantly downregulated oxidase genes from Vyd1515FF were significantly upregulated in the permethrin-resistant population [17]; this shows that these 10 oxidase genes are not significantly involved in either oxamyl or permethrin detoxification/resistance.

All the above data indicated that P450 monooxygenase and esterase genes may be directly involved in the detoxification and decreased susceptibility of TPBs to carbamate insecticide. Two mitochondrial cytochrome c oxidases were significantly upregulated and associated with detoxification pathway and oxamyl resistance. Unexpectedly, GST genes played an insignificant role in oxamyl-treated TPBs.

#### 3.2.2. Hydrolysis Is a Key Detoxification Mechanism in the Survivors of Oxamyl-Treated TPBs

**Figure 2:**
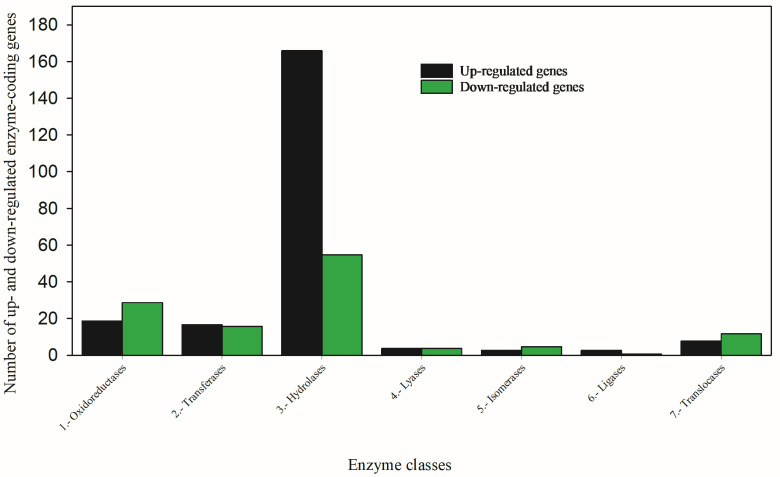
Combined functional analysis revealed seven classes of important metabolic enzymes and numbers of coding genes for the seven enzyme classes.

Functional analysis showed that hydrolase genes were dominantly and substantially upregulated (166 up vs. 55 down) in oxamyl-treated TPBs (Figure 2). Hydrolase is one of the largest groups of structurally related enzymes with diverse catalytic/hydrolytic functions, breaking a chemical bond utilizing water in order to hydrolyze a large molecule into two smaller ones for synthesis, excretion, and energy sources [36]. Some common hydrolases are esterases, lipases, phosphatases, glycosidases, peptidases, and nucleosidases. Carboxylesterases, a subclass of hydrolases (EC 3.1.), specifically cleave and detoxify ester bond-containing carbamate, organophosphate, and pyrethroid insecticides [37]. In oxamyl-treated TPBs, more hydrolase genes (14 without esterases) were significantly upregulated, while only two hydrolase genes were significantly downregulated (Table 1 and Supplementary Table S2). More research is required to understand the functions of hydrolases in the study of insecticide metabolism because members of this group of hydrolytic enzymes play an important role in insecticide poisoning and detoxification [31,38].

#### 3.2.3. Other Enzyme Genes

Several other enzymes may play important roles in metabolic processes and physiological functions, including dehydrogenase, hydrolase, kinase, reductase, synthase, transferase, etc. Their genes also showed different expression patterns. More transferase genes (7) were significantly upregulated in the survivors of oxamyl-treated TPBs and four of the genes were downregulated (Table 1; Supplementary Tables S1 and S2). Kinase, reductase, and synthase showed similar numbers up or downregulated genes. These genes may indirectly influence detoxifying toxicants through mediating biochemistry and physiology after exposure to the insecticide. More investigations are needed to understand whether and why these enzyme gene expressions are concurrently regulated by the oxamyl treatment.

#### 3.2.4. Over Expressions of Digestive Enzyme Genes

Only one polygalacturonase gene was significantly downregulated in oxamyl-treated bugs. The showing of 35 over expressed genes indicated a diversity and complicity of polygalacturonase genes in TPB, similarly existed in a closely related mirid species—the western tarnished plant bug (*L. hesperus*) [39]. Polygalacturonase (EC 3.2.1.15), also known as pectin depolymerase (PG), functions to soften and sweeten fruit during the ripening process. Tarnished plant bugs cause a significant amount of damage to a variety of crops. During feeding, *Lygus* bugs use a piercing mouth part to inject saliva (containing PG for degrading plant cell wall) for facilitating uptake of plant juice [40]. Although polygalacturonases were predominantly upregulated in oxamyl-selected TPB (only one was significantly downregulated), direct association of overexpression of PG genes with oxamyl resistance has not been established in this study and in other researchers’ investigations [41,42]. 

The second most abundantly overexpressed genes are a group of proteolytic enzyme-coding genes, including 24 proteases and 11 trypsins (Table 1 and Supplementary Table S1). Fifteen protease and two trypsin genes were significantly downregulated (Table 1 and Supplementary Table S2), indicating that those genes have been functionally diversified. Protease (peptidase or proteinase) is an enzyme that catalyzes proteolysis and breaks down proteins into smaller polypeptides or single amino acids and spurs the formation of new protein products. [43]. Trypsin is a serine protease, which hydrolyzes proteins by cutting peptide chains mainly at the carboxyl side of the amino acid lysine or arginine [44] (https://en.wikipedia.org/wiki/Trypsin accessed on 31 January 2024). The main function of proteases and trypsins is for food digestion, especially for extra-oral digestion and damage to crops [1]. Proteases and trypsins may mediate activation and degradation of Bt toxin and prompt Bt resistance development in lepidopteran insects [45]. Data from this study showed substantially more upregulated than downregulated protease and trypsin genes in oxamyl-selected bugs (Table 1; Supplementary Tables S1 and S2). Direct evidence of linkage of the digestive enzymes to chemical insecticide resistance has not been well documented. Insecticide resistance is frequently associated with fitness costs in the absence of insecticides. Periodically applying insecticide selection pressure on TPBs may result in reduced viability of the bug colony with decreased expressions of yolk, eggshell, and digestive enzyme genes [15,16]. However, insecticide resistance without fitness cost may be associated with greater accumulation of total proteins and carbohydrates in an insecticide-resistant strain, due to increased digestive efficiency (showing greater serine- and cysteine-proteinases as well as cellulase activities in the resistant strains) [42]. 

### 3.3. Modified Biological Processes, Molecular Functions, and Cellular Components

**Figure 3:**
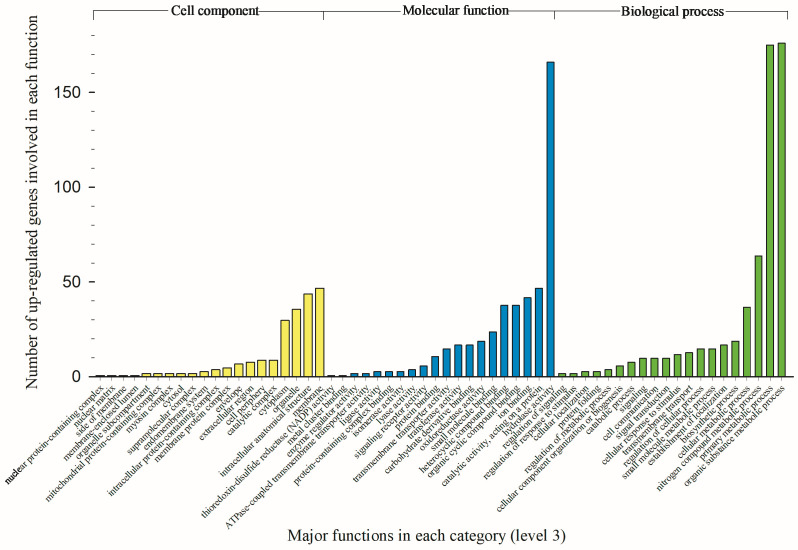
Variable number of upregulated genes associated with each of top 20 functions of cellular components, molecular functions, and biological processes in oxamyl-selected tarnished plant bugs.

**Figure 4:**
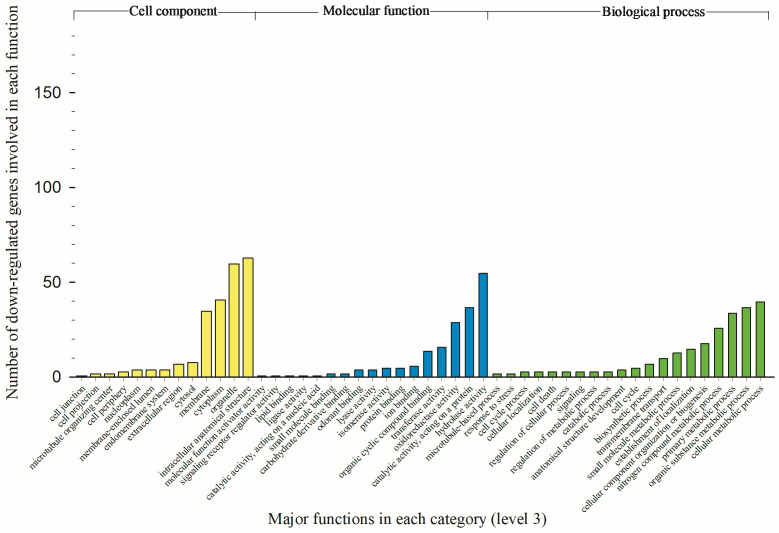
Variable number of downregulated genes associated with each of most available or top 20 functions of cellular components, molecular functions, and biological processes in oxamyl-selected tarnished plant bugs.

Numbers of upregulated genes associated with biological process, molecular function, and cellular component are showed in Figure 3. The total numbers of biological processes, molecular functions and cellular components are 87, 113, and 42, respectively. To show most significantly enhanced functions, only the top 20 functions at level 3 (popular level) are showing in Figure 3. Numbers of downregulated genes associated with biological process, molecular function, and cellular component are showed in Figure 4. The total numbers of biological processes, molecular functions and cellular components are 50, 32, and 23, respectively. Only the top 20 biological processes, 17 molecular functions (available at level 3), and 13 cellular components (available at level 3) are showing in Figure 4. Because of lacking distinctive variation between up and downregulated genes for cellular components, only biological process and molecular function data are presented and discussed below.

For the biological process, 37 genes participated in cellular metabolic process. These 37 genes encoded 10 different enzymes and the most abundantly coded enzymes were kinase, synthase, and dehydrogenase (Figure 3). Sixty-four genes participated in nitrogen compound metabolic process. These 64 genes encoded 13 different enzymes; the most abundantly coded enzymes were protease, trypsin, and kinase. A total of 175 genes participated in primary metabolic process. These 175 genes encoded 18 different enzymes and the most abundantly coded enzymes were polygalacturonases, proteases, and hydrolases (including esterases). A total of 176 genes participated in organic substance metabolic process. These 176 genes encoded 19 different enzymes and the most abundantly coded enzymes were polygalacturonases, proteases, and hydrolases (including esterases). 

For the molecular function, 38 genes participated in organic cyclic compound binding. These 38 genes encoded nine different enzymes and the most abundantly coded enzymes were P450 monooxygenase, kinase, and synthase (Figure 3). Identically, above 38 genes, encoding the same set of enzymes, also participated in heterocyclic compound binding. Forty-two genes participated in ion binding. These 42 genes encoded 14 different enzymes and the most abundantly coded enzymes were P450 monooxygenase, kinase, transferase, and synthase. Forty-seven genes performed catalytic activity (acting on proteins). These 47 genes encoded 10 different enzymes and the most abundantly coded enzymes were protease, trypsin, and kinase. A total of 166 genes conducted hydrolase activity. These 166 genes encoded 15 different enzymes and the most abundantly coded enzymes were polygalacturonase, protease, and hydrolase (including esterases). These data consistently indicated that biological processes and molecular functions were significantly enhanced by over expressions of P450s and hydrolases (esterases), subsequently increasing detoxification of oxamyl in resistant TPB population.

A total of 420 downregulated genes were associated with 13 major biological processes (some genes have multiple roles) in oxamyl-treated TPBs (Figure 4). The cellular metabolic process was the most negatively influenced biological process by 40 genes, coding 15 different enzymes, including dehydrogenase (8), kinase (8), oxidase (6), dismutase (6), and phosphatase (6). The organic substance metabolic process was the second most influenced process by 37 genes, coding 18 enzymes, including kinase, peptidase, and phosphatase of three top influenced enzymes. The primary metabolic process was the third decreased biological process by 34 genes, coding 19 enzymes, including peptidase, kinase, and phosphatase. 

Up to 316 downregulated genes were associated with 10 major molecular functions in oxamyl-treated TPBs (Figure 3). The hydrolase activity was the top negatively affected molecular function by 55 genes, coding 12 enzymes, including peptidase, phosphatase, and carboxypeptidase as the top three reduced enzymes. The catalytic activity (acting on a protein) was the second most influenced molecular function by 37 genes, coding six enzymes, including carboxypeptidases, aminopeptidases, phosphatase, and proteases. Overall, major downregulated genes are not associated with detoxification-related functions in oxamyl-treated TPB population.

### 3.4. Pathway Analyses

Pathway analysis showed that 685 significantly upregulated genes participated in 470 pathways in Vyd1515FF. Based on functional and participation in important biological processes and molecular functions, 24 of the 470 pathways (Table 2) may be directly or indirectly associated in metabolic detoxification and resistance development in TPB. Fifty-two genes, coding seven different enzyme classes, were involved in twenty-four pathways. The top six pathways (with twenty genes coding six enzyme classes) in Table 2 may be directly associated with detoxification. All 11 esterase genes, together with a synthase gene, participated in the drug metabolism (Pathway map ID: ko00983), indicating a primary role in detoxification of carbamate insecticide in TPBs. All 11 esterases belong to type B carboxylesterases (EC 3.1.1.1) that split ester bond to produce an acid and an alcohol in drug metabolism pathway (map00983) of other enzymes. Two esterase genes (LL_6522 and LL_2193) versatilely involve seven additional pathways for neurotransmitter clearance, phase I—functionalization of compound, cholinergic synapse, glycerophospholipid metabolism, LDL clearance, metabolism of angiotensinogen to angiotensins, and synthesis of PC. The cytochrome P450 monooxygenase genes (LL-39, LL_4087, LL_3822, and LL_4510) participated in 12 pathways, including two for detoxification. Oxidase (LL-74) was involved in cytoprotection by HMOX1 and respiratory electron transport, and another oxidase (LL_547), together with synthases (LL_5277 and LL_1710) and a dehydrogenase (LL_2370), was associated with an oxidative phosphorylation (Table 2). 

Analyses showed that 1382 significantly downregulated genes participated in 567 pathways in carbamate selected TPBs. Fifty genes, coding seven different enzymes, were involved in five pathways (Table 3). Oxidative phosphorylation and respiratory electron transport activities might be reduced in Vyda1515FF via reduced gene expressions of oxidase, synthase, dehydrogenase, and ATPase genes. The downregulations of superoxide dismutase and phosphatase genes also reduced detoxification of reactive oxygen species. Noticeably, drug metabolism might be hindered due to the significantly downregulation of a kinase gene. The kinase (EC 2.7.4.6) is a nucleoside-diphosphate kinase, catalyzing phosphorylation of nucleoside diphosphates to nucleoside triphosphates for DNA replication [46]. Understanding why these genes were downregulated in oxamyl-treated TPBs cannot be ignored in further studies.

## 4. Conclusions

After novel microarray global (6688) gene expression analysis, 685 overexpressed genes were sorted. Further functional and pathway analyses obtained 470 pathways and enhanced hydrolase activity (controlled by 166 hydrolase genes) as the most important molecular function. Focusing on 176 upregulated enzyme-coding genes narrowed pathways down from 24 (52 genes) to six metabolic- and detoxification-related pathways, took part via six classes of enzymes coded by 20 genes. Therefore, this study pinpointed 11 ester bond-cutting hydrolases (esterases or carboxylesterases in resistant TPBs), coordinated with two P450s, two oxidases, three synthases, one reductase, and one dehydrogenase, hydrolyzed ester bond-containing oxamyl insecticide. The achievements of this study include at least the following: (1) greatly enhanced knowledge of detoxification and resistance mechanism, (2) established platform to develop molecular tools to monitor and RNA interfeerance to manage resistance in this economically important crop pest, and (3) minimize insecticide contamination and risk to non-target species.

## Figures and Tables

**Table 1:** Altered profiles of gene transcriptions and coded metabolic enzymes in oxamyl-treated TPBs.

176 Upregulated Genes Code 30 Enzymes			120 Downregulated Genes Code 29 Enzymes		
No.	Enzymes	No. of Coding Genes	No.	Enzymes	No. of Coding Genes
1	Esterase	11	1	Esterase	1
2	P450	6 *		P450	0
3	GST	1		GST	0
4	Oxidase	2	2	Oxidase	10
5	Hydrolase	14	3	Hydrolase	2
6	Reductase	6	4	Reductase	1
7	Transferase	7	5	Transferase	4
8	Dehydrogenase	4	6	Dehydrogenase	14
9	Kinase	7	7	Kinase	8
10	Synthase	7	8	Synthase	5
11	ATPase	2	9	ATPase	1
12	Lyase	1	10	Lyase	2
13	Isomerase	1	11	Isomerase	4
	Ligase	0	12	Ligase	3
14	Translocase	1		Translocase	0
15	Thio-Esterase	1		Thio-Esterase	0
	Phospho-Est	0	13	Phospho-Est	1
16	Deoxyribonuclease	1		Deoxyribonuclease	0
17	Phosphatase	3	14	Phosphatase	9
18	lysozyme	2		lysozyme	0
19	Polymerase	1	15	Polymerase	3
	Nucleotidase	0	16	Nucleotidase	1
20	Amidase	1		Amidase	0
	Chitinase	0	17	Chitinase	1
	Ovochymase	0	18	Ovochymase	1
21	Amylase	1	19	Amylase	2
22	Carboxypeptidase	4	20	Carboxypeptidase	6
23	Cathepsin	3	21	Cathepsin	1
	CysProtease	0	22	CysProtease	2
	Dismutase	0	23	Dismutase	6
24	Glucosidase	4	24	Glucosidase	1
25	Lipase	13	25	Lipase	2
26	Peptidase	1	26	Peptidase	11
27	Polygalacturonase	35	27	Polygalacturonase	1
28	Protease	24	28	Protease	15
29	Trypsin	11	29	Trypsin	2
30	Transcriptase	1		Transcriptase	0

* LL-39: similar to P450 Cyp46a1 [*Apolygus lucorum*], LL_4510: similar to P450 CYP395H1 [*Apolygus lucorum*], LL_4087: similar to P450 CYP6a14 [*Cimex lectularius*], LL_3822: similar to P450 CYP4C1 [*Cimex lectularius*], LL_3359: similar to P450 CYP395H1 [*Apolygus lucorum*], and P450 6a14 [*Cimex lectularius*], LL_4711: similar to P450 CYP6a14 [*Halyomorpha halys*].

**Table 2:** Upregulated genes and their major roles in metabolic pathways in Vyda1515FF resistant population (analyzed using OmicsBox Kegg/Eggnog pathway analysis).

Pathway	Pathway ID	Seqs	Gene Sequences ID	Coded Enzyme
Drug metabolism—other enzymes	ko00983	11	LL_2770; LL-223; LL_5104; LL_6522; LL_2244; LL_2193; LL_2639; LL_2508; LL_1233; LL_2520; LL_2600	Esterase
		1	LL_2533	Synthase
Xenobiotics	R-MMU-211981	1	LL-39 *	P450
	R-GGA-211981	1	LL_4087 *	P450
Detoxification of reactive oxygen species	R-DME-3299685	1	LL_3600	Reductase
Aflatoxin activation and detoxification	R-GGA-5423646	2	LL_4087; LL-39	P450
Oxidative phosphorylation	ko00190	1	LL_547	Oxidase
		2	LL_5277; LL_1710	Synthase
		1	LL_2370	Dehydrogenase
Respiratory electron transport	R-DME-611105	1	LL_74	Oxidase
Neurotransmitter clearance	R-DME-112311	1	LL_6522	Esterase
Eicosanoids	R-RNO-211979	1	LL_4510	P450
Arachidonic acid metabolism	ko00590	1	LL_4510	P450
		3	LL_5131; LL_5648; LL_4347	Reductase
Biosynthesis of maresin-like SPMs	R-MMU-9027307	1	LL-39	P450
	R-GGA-9027307	1	LL_4087	P450
Endogenous sterols	R-MMU-211976	1	LL_3822	P450
Synthesis of leukotrienes (LT) and eoxins (EX)	R-RNO-2142691	1	LL_4510	P450
Phase I—functionalization of compounds	R-HSA-211945	1	LL_2193	Esterase
	R-DME-211945	1	LL_6522	Esterase
	R-SSC-211945	2	LL_5131; LL_4347	Reductase
	R-CFA-211945	1	LL_5648	Reductase
Cholinergic synapse	ko04725	2	LL_6522; LL_2193	Esterase
Cytoprotection by HMOX1	R-DME-9707564	1	LL_74	Oxidase
Fatty acids	R-RNO-211935	1	LL_4510	P450
Glycerophospholipid metabolism	ko00564	2	LL_6522; LL_2193	Esterase
LDL clearance	R-DME-8964038	1	LL_6522	Esterase
Metabolism of angiotensinogen to angiotensins	R-HSA-2022377	1	LL_2193	Esterase
Miscellaneous substrates	R-RNO-211958	1	LL_4510	P450
	R-GGA-211958	1	LL_4087	P450
Platelet activation	ko04611	1	LL_4510	P450
Synthesis of (16–20)-hydroxyeicosatetraenoic acids (HETE)	R-RNO-2142816	1	LL_4510	P450
Synthesis of PC	R-DME-1483191	1	LL_6522	Esterase
	R-MMU-1483191	1	LL_2133	kinase
The canonica+6:42l retinoid cycle in rods (twilight vision)	R-MMU-2453902	1	LL_3822	P450

* LL-39: similar to P450 Cyp46a1 [*Apolygus lucorum*], LL_4087: similar to P450 CYP6a14 [*Cimex lectularius*].

**Table 3:** Downregulated genes and their major roles in metabolic pathways (analyzed using OmicsBox Kegg/Eggnog pathway analysis).

Pathway	Pathway ID	Seqs	cDNA Sequence ID	Coded Enzymes
Oxidative phosphorylation	ko00190	1	LL_2757	ATPase
		2	LL_725; LL_1114	Cytochrome b
		4	LL_1370; LL_1608; LL_1489; LL_2446	Dehydrogenase
		2	LL_6245; LL_3052	Hypothetical protein
		9	LL_119; LL_128; LL_3927; LL_222; LL_3845; LL_791; LL_5019; LL_317; LL_2260	Oxidase
		4	LL_1620; LL_1695; LL_2225; LL_6173	Synthase
Respiratory electron transport	R-DME-611105	12	LL_725; LL_317; LL_119; LL_128; LL_2260; LL_3927; LL_222; LL_1114; LL_3845; LL_1531; LL_791; LL_5019	Oxidase
TP53 regulates metabolic genes	R-DME-5628897	9	LL_317; LL_119; LL_128; LL_3927; LL_222; LL_3845; LL_1531; LL_791; LL_5019	Oxidase
Detoxification of reactive oxygen species	R-DRE-3299685	4	LL_2080; LL-34; LL_6623; LL_3138	Superoxide dismutase
		2	LL_1663; LL_164	Phosphatase
Drug metabolism—other enzymes	ko00983	1	LL_1672	Kinase

## Data Availability

The data that support the findings of this study are available on request from the corresponding author, [Y.-C.Z.].

## Supplementary Materials

The following supporting information can be downloaded at: https://www.mdpi.com/article/10.3390/toxics12030188/s1, Table S1. Identification of 176 metabolic enzyme-coding genes showing significantly upregulated (≥2-fold) gene expressions in Vyda1515FF resistant population using microarrays and analyzed with ArrayStar and OmicsBox protocols (www.biobam.com, accessed on 31 January 2024); Table S2. Identification of 120 metabolic enzyme-coding genes showing significantly downregulated (≥2-fold) gene expressions in Vyda1515FF resistant population using microarrays and analyzed with ArrayStar and OmicsBox protocols (www.biobam.com, accessed on 31 January 2024).
