# Supplementary material for: Microarray and Functional Pathway Analyses Revealed Significantly Elevated Gene Expressions Associated with Metabolic Resistance to Oxamyl (Vydate) in Lygus lineolaris
Source: Toxics. 2024 Feb 28;12(3):188. doi: 10.3390/toxics12030188 (PMC10973988; doi:10.3390/toxics12030188)
Supplement: Supplementary file 1 [file toxics-12-00188-s001.zip › toxics-2876981-supplementary.pdf]

toxics

Supplementary Table S1. Identification of 176 metabolic-enzyme-coding genes showing significantly up-regulated (≥2-fold) gene expressions in Vyda1515FF resistant population using microarrays and analyzed with ArrayStar and OmicsBox protocols (www.biobam.com)

SEQ_ID	Seq Length (bp)	Coded Enzymes	Gene Expression (Fold)	SEQ_ID	Seq Length (bp)	Coded Enzymes	Gene Expression (Fold)
LL_2338	720	Amidase	2.4030	LL_5516	507	polygalacturonase	9.1670
LL_4739	426	Amylase	2.3990	LL_6079	627	polygalacturonase	8.9470
LL_4684	707	ATPase	5.2200	LL_3453	255	polygalacturonase	7.9890
LL_3896	741	ATPase	2.6660	LL_5437	184	polygalacturonase	7.6820
LL_3508	139	Carboxypeptidase	7.8250	LL_3626	443	polygalacturonase	6.9770
LL-190	564	Carboxypeptidase	5.3470	LL_999	871	polygalacturonase	6.4650
LL_1451	446	Carboxypeptidase	3.6590	LL_2546	552	polygalacturonase	6.1270
LL_2428	485	Carboxypeptidase	2.0390	LL_426	590	polygalacturonase	5.9570
LL_5197	626	Cathepsin	6.5780	LL_1732	754	polygalacturonase	5.8240
LL_1851	692	Cathepsin	2.9010	LL_1124	192	polygalacturonase	5.7320
LL_194	528	Cathepsin	2.6620	LL_1727	543	polygalacturonase	5.5020
LL_61	935	Dehydrogenase	2.3630	LL-166	266	polygalacturonase	5.1710
LL_4446	680	Dehydrogenase	2.0920	LL_65	1184	polygalacturonase	4.7360
LL_2340	753	Dehydrogenase	2.0860	LL_51	648	polygalacturonase	4.5350
LL_2370	488	Dehydrogenase	2.0610	LL_2251	602	polygalacturonase	4.4860
LL_2916	830	deoxyribonuclease	5.2670	LL_3310	535	polygalacturonase	4.4370
LL_2508	864	Esterase	15.7780	LL_3956	564	polygalacturonase	4.3760
LL_2193	811	Esterase	13.0150	LL_1464	351	polygalacturonase	4.1850
LL_6522	772	Esterase	8.7520	LL_271	1194	polygalacturonase	4.0080
LL-223	668	Esterase	7.8750	LL_4139	505	polygalacturonase	3.7370
LL_2244	471	Esterase	5.1860	LL_2680	733	polygalacturonase	3.5660
LL_1233	317	Esterase	3.4200	LL_2594	463	polygalacturonase	3.4080
LL_2770	368	Esterase	3.2080	LL_1904	419	polygalacturonase	3.2690
LL_2520	557	Esterase	2.5240	LL_450	842	polygalacturonase	3.1630
LL_2639	443	Esterase	2.4030	LL_2157	383	polygalacturonase	2.9670
LL_5104	610	Esterase	2.3310	LL_991	191	polygalacturonase	2.7350
LL_2600	557	Esterase	2.1050	LL_5374	421	polygalacturonase	2.5670
LL-64	697	Glucosidase	6.3460	LL_516	803	polygalacturonase	2.4110
LL_3101	702	Glucosidase	4.1540	LL-139	573	polygalacturonase	2.0550
LL_2556	215	Glucosidase	3.8610	LL_476	747	polymerase	2.4980
LL_5652	890	Glucosidase	2.0080	LL_2022	772	Protease	9.3090
LL_892	412	GST	2.2910	LL_978	799	Protease	7.4880
LL-81	655	Hydrolase	9.3560	LL-291	738	Protease	5.3950
LL_4154	473	Hydrolase	8.8460	LL_3525	469	Protease	4.9100
LL_3690	672	Hydrolase	8.6750	LL_2070	605	Protease	4.6670
LL-559	659	Hydrolase	7.9050	LL_2229	438	Protease	4.4580
LL_2938	772	Hydrolase	7.5680	LL_2925	432	Protease	4.3200
LL_2937	653	Hydrolase	6.9270	LL_2062	714	Protease	4.1640
LL_6356	691	Hydrolase	6.1860	LL_4695	625	Protease	4.0010

LL_4801	662	Hydrolase	4.1020	LL_5942	176	Protease	3.6610
LL_3085	631	Hydrolase	3.9830	LL_1015	554	Protease	3.6110
LL_1637	814	Hydrolase	3.4210	LL-593	348	Protease	3.5910
LL-134	676	Hydrolase	2.1330	LL_436	648	Protease	3.5570
LL_2567	841	Hydrolase	2.1110	LL_1481	323	Protease	3.2480
LL_6252	317	Hydrolase	2.1040	LL_5188	657	Protease	2.9690
LL_5529	542	Hydrolase	2.0210	LL_4752	451	Protease	2.8560
LL_4630	325	isomerase	4.7160	LL-487	659	Protease	2.6970
LL_2649	733	Kinase	5.8490	LL_255	614	Protease	2.6880
LL_2133	473	Kinase	2.8290	LL_481	422	Protease	2.6500
LL-646	725	Kinase	2.2960	LL_5388	382	Protease	2.4100
LL_1772	798	Kinase	2.0810	LL_1837	710	Protease	2.3880
LL_3756	853	Kinase	2.0400	LL-400	507	Protease	2.3860
LL-616	617	Kinase	2.0080	LL_3669	430	Protease	2.3790
LL_5168	387	Kinase	2.0020	LL_2675	394	Protease	2.1950
LL_749	757	Lipase	18.0010	LL_3600	591	Reductase	4.9080
LL_4384	428	Lipase	17.6540	LL_3143	557	Reductase	4.8940
LL_4161	383	Lipase	16.5670	LL_5131	572	Reductase	3.7940
LL_2761	715	Lipase	16.1860	LL_4347	499	Reductase	2.2790
LL_1758	600	Lipase	11.7260	LL_5648	333	Reductase	2.1510
LL-594	676	Lipase	10.6990	LL_3192	636	Reductase	2.0020
LL_5941	647	Lipase	9.8710	LL_2533	678	Synthase	2.7510
LL_3736	614	Lipase	5.4730	LL_6333	410	Synthase	2.5240
LL_2994	347	Lipase	3.4960	LL_5277	803	Synthase	2.2940
LL_2151	676	Lipase	2.7070	LL_1710	446	Synthase	2.1560
LL_1954	758	Lipase	2.5490	LL_3038	652	Synthase	2.1220
LL_2003	508	Lipase	2.1370	LL_4018	395	Synthetase	4.0310
LL_6274	577	Lipase	2.0070	LL_4337	616	Synthetase	2.3090
LL_2239	670	Lyase	3.2600	LL_1860	834	thio-Esterase	2.3060
LL_4073	454	lysozyme	4.3610	LL_5732	421	transcriptase	8.3860
LL-749	601	lysozyme	2.6020	LL_2760	769	Transferase	3.1540
LL_547	684	Oxidase	3.9260	LL_579	774	Transferase	2.4590
LL_74	546	Oxidase	2.7590	LL_5562	391	Transferase	2.2670
LL_4510	676	P450	4.7350	LL-318	580	Transferase	2.2200
LL_3359	640	P450	4.1370	LL_5629	417	Transferase	2.1350
LL-39	599	P450	3.7780	LL_5988	638	Transferase	2.0250
LL_3822	852	P450	3.4730	LL-394	332	Transferase	2.0010
LL_4087	628	P450	3.0480	LL_704	582	Translocase	2.7450
LL_4711	289	P450	2.1130	LL_2652	148	Trypsin	6.3470
LL_5661	806	Peptidase	2.2500	LL_3149	297	Trypsin	4.9900
LL_6649	815	Phosphatase	3.5680	LL_1745	226	Trypsin	4.5060
LL_4408	543	Phosphatase	3.2690	LL_2286	832	Trypsin	4.5030
LL_1856	647	Phosphatase	2.2140	LL-598	499	Trypsin	4.2530
LL_4791	231	polygalacturonase	14.7200	LL_5354	307	Trypsin	4.0150
LL_2654	485	polygalacturonase	12.1280	LL_6160	504	Trypsin	3.7230
LL_1641	782	polygalacturonase	10.8350	LL_4275	492	Trypsin	3.5370
LL-293	600	polygalacturonase	10.1250	LL_641	855	Trypsin	3.3320

LL_660	531	polygalacturonase	9.9010		LL_504	914	Trypsin	3.3000
LL_5763	465	polygalacturonase	9.4770		LL_3705	384	Trypsin	2.6090

Supplementary Table S2. Identification of 120 metabolic-enzyme-coding genes showing significantly down-regulated (≥2-fold) gene expressions in Vyda1515FF resistant population using microarrays and analyzed with ArrayStar and OmicsBox protocols (www.biobam.com)

**SEQ_ID**	**Seq Length (bp)**	**Coded Enzymes**	**Gene Expression (Fold)**		**SEQ_ID**	**Seq Length (bp)**	**Coded Enzymes**	**Gene Expression (Fold)**
LL_1494	355	Amylase	2.4310		LL-567	549	Oxidase	3.4870
LL_2588	600	Amylase	2.2150		LL_5776	615	Oxidase	2.9380
LL_2757	520	ATPase	2.5360		LL_222	1187	Oxidase	2.8640
LL_4865	428	Carboxypeptidase	4.9430		LL_5314	168	Oxidase	2.8500
LL_868	480	Carboxypeptidase	3.4680		LL_3845	457	Oxidase	2.4720
LL_4155	447	Carboxypeptidase	2.6540		LL_791	562	Oxidase	2.3720
LL_305	623	Carboxypeptidase	2.4240		LL_119	386	Oxidase	2.0770
LL_4429	458	Carboxypeptidase	2.3410		LL_128	314	Oxidase	2.0470
LL_3947	695	Carboxypeptidase	2.0600		LL_5055	493	Peptidase	5.3270
LL_5512	379	Cathepsin	2.5370		LL-617	687	Peptidase	3.3810
LL_6405	641	Chitinase	4.6220		LL_5306	524	Peptidase	3.3520
LL_3456	313	Cysteine peptidase	2.3040		LL-751	323	Peptidase	2.8530
LL_3856	658	Cysteine protease	2.0620		LL_2135	531	Peptidase	2.5400
LL_3901	397	Dehydrogenase	3.6890		LL_4592	575	Peptidase	2.4630
LL_2446	381	Dehydrogenase	3.4350		LL_3193	662	Peptidase	2.4300
LL_1432	286	Dehydrogenase	3.0660		LL_4476	520	Peptidase	2.4090
LL_4362	173	Dehydrogenase	3.0600		LL-252	558	Peptidase	2.3890
LL_1608	316	Dehydrogenase	2.9720		LL_2086	833	Peptidase	2.3040
LL_5491	419	Dehydrogenase	2.9580		LL_6093	718	Peptidase	2.2200
LL-566	715	Dehydrogenase	2.6850		LL_1181	298	Phosphatase	4.8560
LL_2979	655	Dehydrogenase	2.6830		LL_2305	433	Phosphatase	3.4660
LL-38	465	Dehydrogenase	2.4090		LL_160	594	Phosphatase	3.1370
LL_5093	606	Dehydrogenase	2.3860		LL_1663	471	Phosphatase	2.9950
LL-666	466	Dehydrogenase	2.2960		LL_5734	775	Phosphatase	2.9260
LL_1489	152	Dehydrogenase	2.2290		LL-271	561	Phosphatase	2.8170
LL-738	345	Dehydrogenase	2.1350		LL_6134	337	Phosphatase	2.7230
LL_1370	299	Dehydrogenase	2.1310		LL_606	602	Phosphatase	2.3320
LL_2080	652	Dismutase	2.7750		LL-199	551	Phosphatase	2.2980
LL-552	358	Dismutase	2.7480		LL_3784	573	Phospho-Est	3.8660
LL_6623	606	Dismutase	2.7100		LL_3689	794	Polygalacturonase	12.3040
LL-34	524	Dismutase	2.7000		LL_4311	805	Polymerase	2.4370
LL_164	626	Dismutase	2.6620		LL_392	641	Polymerase	2.3520
LL_3138	603	Dismutase	2.0790		LL_6657	561	Polymerase	2.1570
LL-227	422	Esterase	2.8260		LL_3996	338	Protease	6.6130
LL_1217	288	Glucosidase	3.6870		LL_1957	401	Protease	5.8850
LL_1592	389	Hydrolase	2.5000		LL_2335	724	Protease	3.7610
LL_4560	611	Hydrolase	2.0020		LL_4947	524	Protease	3.4080
LL_5561	649	Isomerase	2.8800		LL-378	436	Protease	3.3200
LL_4433	630	Isomerase	2.6580		LL_3660	321	Protease	2.9420
LL_4017	486	Isomerase	2.5920		LL-156	563	Protease	2.8560

LL_5692	716	Isomerase	2.1270		LL_1995	509	Protease	2.3260
LL-369	511	Kinase	3.7230		LL_1017	814	Protease	2.2100
LL_1672	212	Kinase	2.7080		LL_2667	643	Protease	2.1430
LL_5371	592	Kinase	2.6750		LL-440	562	Protease	2.1390
LL_6322	452	Kinase	2.5370		LL_3934	741	Protease	2.0900
LL_1805	812	Kinase	2.2750		LL_5085	593	Protease	2.0640
LL_1272	297	Kinase	2.2270		LL_6089	612	Protease	2.0510
LL_4052	632	Kinase	2.1790		LL-317	696	Protease	2.0460
LL_4079	599	Kinase	2.1040		LL_5391	501	Reductase	2.3480
LL_3394	642	Ligase	3.0450		LL_4913	421	Synthase	3.1230
LL-777	608	Ligase	2.3520		LL_1695	524	Synthase	3.2520
LL_5990	742	Ligase	2.2760		LL_2225	580	Synthase	2.6060
LL_4363	503	Lipase	2.5240		LL_6173	786	Synthase	2.1330
LL-220	612	Lipase	2.4880		LL_1620	677	Synthase	2.0850
LL_238	729	Lyase	2.3090		LL_2365	515	Transferase	3.3850
LL_5148	549	Lyase	2.1680		LL_6423	783	Transferase	3.0700
LL_4424	518	Nucleotidase	2.7550		LL_179	845	Transferase	2.9590
LL_4473	671	Ovochymase	3.3050		LL_1722	543	Transferase	2.7040
LL_5019	577	Oxidase	3.7850		LL-523	573	Trypsin	5.8520
LL_3927	451	Oxidase	4.2510		LL_3618	217	Trypsin	2.2840
